# Developing community based rehabilitation for cancer survivors: organizing for coordination and coherence in practice

**DOI:** 10.1186/1472-6963-13-339

**Published:** 2013-09-02

**Authors:** Karen la Cour, Malcolm P Cutchin

**Affiliations:** 1 Research Initiative of Activity Studies and Occupational Therapy, Research Unit Health, Man and Society, Institute of Public Health, University of Southern Denmark, J. B. Winsløws Vej 9, 5000 Odense, Denmark; 2 Department of Health Care Sciences, Eugene Applebaum College of Pharmacy and Health Sciences Wayne State University, Detroit, USA

## Abstract

**Background:**

Increasing incidences of cancer combined with prolonged survival have raised the need for developing community based rehabilitation. The objectives of the analysis were to describe and interpret the key issues related to coordination and coherence of community-based cancer rehabilitation in Denmark and to provide insights relevant for other contexts.

**Methods:**

Twenty-seven rehabilitation managers across 15 municipalities in Denmark comprised the sample. The study was designed with a combination of data collection methods including questionnaires, individual interviews, and focus groups. A Grounded Theory approach was used to analyze the data.

**Results:**

A lack of shared cultures among health care providers and systems of delivery was a primary barrier to collaboration which was essential for establishing coordination of care. Formal multidisciplinary steering committees, team-based organization, and informal relationships were fundamental for developing coordination and coherence.

**Conclusions:**

Coordination and coherence in community-based rehabilitation relies on increased collaboration, which may best be optimized by use of shared frameworks within and across systems. Results highlight the challenges faced in practical implementation of community rehabilitation and point to possible strategies for its enhancement.

## Background

Cancer survivors can be defined as persons ‘living with a cancer diagnosis following primary cancer treatment for cancer through the end of life’ [1]. The number of people globally who survive or live for extensive periods of time after a cancer diagnosis is growing [2]. In Denmark there are more than 300,000 cancer survivors out of a population of 5.4 million people. Increasing survival rates result in growing requests for services to support cancer patients, such as those making the transition from hospital care to community living. This situation has in turn increased demands for community-based rehabilitation and survivor care. Moreover, cancer survivors may be subject to sequelae including physiological and psychological after-effects of cancer and treatment that can profoundly influence health status as well as quality of life [3,4].

Consequently, coordination and quality of follow-up care are needed for rehabilitation after hospital-based interventions. Although community-based cancer rehabilitation is being developed around the world, we have yet to discover what key issues are being encountered in implementation and how they might best be solved. More specifically, we need to identify how community-based rehabilitation can be efficiently organized and how adequate coordination and coherence between health care providers and across sectors can be optimized in practice. This paper begins to address these gaps based on a national study from Denmark utilizing data from the perspective of the health care professionals involved in community-based cancer rehabilitation.

In recent years there has been an increasing international interest among health care administrators and providers of cancer care in developing community-based rehabilitation [3,5,6]. In particular, a focus on coordination between health-care providers has been identified as essential for coherence of the cancer survivors’ rehabilitation course, yet significant challenges remain [7]. Cancer rehabilitation is a complex healthcare activity that consists of multiple services involving several professionals to provide biomedical as well as psychosocial interventions [8,9]. In order to provide such services it is necessary to develop coordination and coherence throughout the course of rehabilitation. In this context, coordination refers to the integration of interdisciplinary rehabilitation services whether delivered by an array of providers at the community level or across levels of care such as from a hospital to the community. Coherence in this study refers to the quality of alignment among the various services provided to the individual cancer survivor.

While prior research has addressed the organization and provision of cancer rehabilitation and survivor care [4,10,11], it rarely has included viewpoints from practitioners in the multiple disciplines that provide rehabilitation for cancer survivors on a day to day basis. Hence, exploration and descriptions of the actual challenges of implementing interdisciplinary cancer rehabilitation are needed.

In Denmark, municipalities and local communities have a long history of providing services for people who are limited in their ability to manage on their own in their home environment. Community-based rehabilitation of cancer survivors has, however, not been common. Following changes in the Danish Healthcare Law in 2007, we witnessed an increasing focus on community-based rehabilitation for people surviving cancer. This legal act placed the primary responsibility for providing rehabilitation onto the municipalities, including the obligation to provide coordinated and coherent rehabilitation care. With a focus on coordination and coherence, the Danish healthcare system emphasized securing coordination of services among disciplines and across sectors in rehabilitation services as well as coherence in the rehabilitation trajectory.

In order to meet these demands, funding was given to 11 projects in 15 municipalities across Denmark to explore how community-based rehabilitation for cancer survivors could be optimally developed with regard to its organization and services. Three areas of rehabilitation were prioritized: (1) coordination and coherence in the transition from hospital to community based services, (2) physical activity, and (3) returning to life, including coping with changed life conditions and the return to work. The first of these areas focused on organizational aspects of the provision of rehabilitation, while the other two pointed to specific intervention areas in relation to the cancer survivors.

We focus on the first area in this paper. The objective of the study was to explore and describe the key issues, such as structural as well as inter-professional challenges related to coordination and coherence of community-based cancer rehabilitation in the Danish case. Taking into account that the communities in this study did not have any established professional and collaborative patterns for this task, we chose to focus on the perspectives of rehabilitation managers involved in the organization and delivery of care. A subsequent goal of the analysis was to provide insights that might be relevant not only for cancer rehabilitation in Denmark but in other contexts as well.

## Methods

The study was part of a large multicenter program aiming to develop community-based rehabilitation in Denmark as well as to evaluate that development and the resulting services. In order to explore and understand ways in which such a program can be implemented, we employed a qualitative methodology to gain access to social processes involved in organization and collaboration in rehabilitation. We employed a combination of methods to generate data, and consistent with such a problem, we employed principles of Grounded Theory to analyze the data [12].

### Sample

Participants comprise 27 Rehabilitation Managers (RMs) who were hired by the 11 projects to manage and develop cancer rehabilitation in 15 diverse municipalities across Denmark. All RMs were included. RMs in this study are similar to what are often called case-managers or patient navigators. RMs coordinates rehabilitation-services and guide cancer survivors through the complex and multiple services needed. RMs were responsible for developing and organizing the delivery of community-based cancer rehabilitation in the municipalities. The RMs were distributed among the following professions: 12 nurses, 5 social workers, 3 medical doctors (MDs), 2 physiotherapists, 1 occupational therapist, 1 anthropologist, and 3 healthcare administrators.

### Data collection

Data were gathered using a combination of methods including questionnaires with closed and open-ended questions, semi-structured interviews, and focus-group interviews [13,14]. The overall approach was to develop knowledge about central issues, such as structural barriers and inter-professional challenges, probe those issues to develop greater depth of understanding, and to then expand and validate that understanding. First, descriptive data on the individual projects were collected via a questionnaire regarding local practices mailed to the 27 rehabilitation-managers (RMs). The instrument included items pertaining to the organization and content of the rehabilitation programs in each municipality, including open-ended questions about how the programs were run. For example, questions included: From where and how are cancer survivors recruited? How is rehabilitation delivery and collaboration among service providers organized? All 27 RMs returned a completed questionnaire for a 100 percent response rate. An initial analysis of the closed- and open-ended responses was conducted to develop initial understandings of the programs and key issues. Those understandings were investigated further through follow-up interviews and focus group interviews.

Using insights gleaned from the questionnaire data, two researchers (including the first author) conducted follow-up interviews with the RMs in each of the 15 communities. A generic interview guide based on the questionnaire insights was used. That guide included topics such as specification of procedures for inclusion of cancer survivors, organization and type of rehabilitation interventions. The interviews served to probe more deeply into the processes and contexts of organizational and delivery challenges in the projects. The interviews took place in the communities where the RMs were working. Each interview lasted between 1 – 2 hours. All interviews were recorded.

To further expand RMs’ different perspectives and validate data and insights, focus group interviews were employed [15]. All RMs were invited to join focus group interviews that were conducted at a geographically central site in Denmark. Twenty-one focus groups were conducted over a two-year period. The focus group interviews were organized as part of day-long workshops. On the mornings of the workshops, the 27 rehabilitation RMs were provided with an expert lecture relevant for cancer-rehabilitation. The lectures were designed to stimulate thinking and increase knowledge of appropriate issues, for example, contemporary taxonomies and methods used in the field, an introduction to the International Classification system of Functioning (ICF), and tools to identify rehabilitation needs and methods for evaluation [16,17]. After the lecture, participants were divided into 3 groups for focus-group interviews [15]. Composition of the groups varied from one workshop to another, with 7 to 9 participants in each group, depending on number of RMs who attended that day. The focus-group discussions were chaired by members of the team established to support the local projects and conduct project evaluation. The purpose of the focus-groups was twofold: (1) to elicit RM opinions and insights about their specific experiences of developing community-based rehabilitation with attention to areas of disagreements and consensus, and (2) to facilitate knowledge exchange among RMs [14,15]. For example, participants were encouraged to exchange experiences and share opinions about how to best organize community-based cancer rehabilitation in order to coordinate and create a coherent rehabilitation process for cancer survivors. Although coordination and collaboration were not the main subjects of more than one workshop, organizational and collaborative issues were raised by RMs during all focus group discussions. In line with ideas of Grounded Theory, the framework for each focus-group interview was continuously adapted as new insights were gained from previous data collection and ongoing analysis. The focus group interviews were recorded and the focus-group leaders from the evaluation team wrote memos about each group-session.

### Data analysis

The qualitative data from questionnaires and the semi-structured interview recordings were transcribed verbatim. Based on careful listening to each focus group recording combined with the written memos from each session the lead evaluator (first author) wrote out extensive summaries of the focus group interviews including quotes of discussion parts central to the study objective. All qualitative data were analyzed using a constant comparative method according to the chosen Grounded Theory approach [12]. Text from questionnaires, individual interviews, and focus group interviews were read to achieve a thorough understanding of the RM’s experiences of developing community-based rehabilitation. Open coding was performed whereby codes were assigned to the data based on a line-by-line reading. Thereafter, the initial codes were compared in a back and forth process to test, consolidate, and refine emerging categories of codes. Throughout the analysis process, codes and categories were compared and related to each other to clarify and solidify primary categories. Analysis of the data was conducted independently by the first author. Subsequently, the findings were examined by another, experienced researcher. RMs also reviewed the findings and provided feedback that was used to hone the understanding. This last process lead to minor modifications of codes and categories to finalize the analysis.

### Ethics

The Study complied with the Helsinki Declaration, the Ethical committee System and was approved by the Danish Data Protection Agency identification number 2008-41-2417. Written consent was obtained from all study participants.

## Results

The three main conceptual categories that emerged from the analysis were: (1) cultures that govern coordination, (2) insufficient pathways and collaborative keys to rehabilitation, and (3) advantages of team-based rehabilitation (see Overview of results). We present the categories below complemented by pertinent details and examples that help to contextualize the findings. To ensure anonymity all names of places and individuals are pseudonyms.

### Overview of results

#### Cultures that govern coordination

Cancer care traditions by context and people.

Relationships and steering committees as facilitators.

#### Insufficient pathways and collaborative keys to rehabilitation

Barriers for community-based cancer rehabilitation.

Strategies for equal inclusion.

#### Advantages of team-based rehabilitation

Two ways of organizing rehabilitation-management.

### Cultures that govern coordination

The analysis showed that several cultural dimensions of the rehabilitation process were of importance to intervention coordination. In particular we identified that culture expressed through discourses and traditions practiced in given contexts and by people of specific professions govern coordination. Collaboration was identified to be challenging interpersonally among healthcare providers as well as structurally between sectors within a given Municipality as well as with sectors and collaborating parties outside the individual Municipality. In addition, we found that coordination can be facilitated by informal and formal relationships within the care sector.

### Cancer care traditions by contexts and people

According to the RMs in our study, collaboration is fundamental to the coordination of multi-modal therapies and specialized treatment. Because the projects were a new initiative, there were no traditions for collaboration about cancer rehabilitation in the municipalities involved. The RMs reported that there were no pre-established pathways of communication that they could follow. Nor did they find any guideline for coordinating the various interventions that cancer survivors could be in need of. In this regard it should be noted that, although cure was expected for the cancer survivors referred to the project of community-based rehabilitation, the citizens who participated in the rehabilitation programs were in varying stages of cancer diagnosis. The majority were suffering from a variety of physical and emotional problems that needed close collaboration and exchange of knowledge between hospital staff, social workers, general practitioners, dieticians, physiotherapists, occupational therapists, etc.

As per pathways and traditions of care, within the individual communities as well as in the participating institutions (organizations, hospital and community) there was no traditional structure for how rehabilitation should be provided or what the community rehabilitation should entail. For instance, the RMs pointed to a variety of different foci of cancer rehabilitation (pain control, exercise, diet, social assistance, return to work, etc.) among the different disciplines involved. This fragmentation of disciplinary focus proved to be a major obstacle for collaboration as the basis for developing coordinated and coherent rehabilitation services to the individual citizen.

A particular type of situation was highlighted by the RMs that different discourses dominate within different delivery subsystems and disciplines. For example a biomedical discourse was identified to dominate in hospitals contexts whereas a psychosocial discourse characterized the contexts of community based services. In addition, different approaches to rehabilitation were identified across departments in a municipality. One of the RMs, Maria, shared that:

In the community, where I am based, in the social welfare department we have great results with cancer survivors who have problems with returning to work, whereas health oriented needs are not met to the same extent.

This example indicates how the particular context and distribution of labor (and professional expertise) within a local cancer rehabilitation system creates unequal outcomes. Local cultures of cancer rehabilitation emerged based on who was there to provide care and from which departments influencing the discourse of care. It could be argued that a hierarchical structure among professions caused poor relations at times, or more simply it could be interpreted that communication is smoother within a profession. In addition to the challenges of dividing discourses, decisions about the content of cancer rehabilitation were complicated further as many of the required services had not been subject to sufficient and stringent research. Such lack of evidence was a continuous challenge for the RMs who reported that the lack of documentation of specific interventions made it difficult for them to build rehabilitation on solid ground.

### Relationships and steering committees as facilitators

Personal relations among various parties involved in the rehabilitation process were central to good care. Relationships and continuous communication were noted by our participants as important for establishing and maintaining good collaboration, especially across delivery borders (between institutions). In turn, collaboration was suggested by RMs as essential for coordination and coherence in the provision of rehabilitation. Repeated visits by the RM to hospitals and other community administrative sites, and thus visibility to other involved parties were part of this process. In addition, cross-disciplinary steering committees proved to be of utmost importance in overcoming potential problems to coherence. As one of the RMs said:

Many of us whom are now employed at the community rehabilitation centre have previously been working at the local hospital from where we recruit cancer clients and survivors. That means we know each other across systems and it makes it easier to just pass by the hospital wards and remind them to refer patients to us. In extension we have held several information meetings and lectures which in turn make the staff from the hospital come to us and benefit from the competencies we offer (Jesper).

As seen in this example, community-based rehabilitation projects that recruited staff from hospitals found that this strategy eased coordination of rehabilitation, since the staff already had an established network within the hospital. Informal and personal relationships and networks across delivery systems were identified as the strongest vehicle for collaboration as a basis for coordinating services and creating coherence in patients’ rehabilitation course.

Establishing steering committees that included representatives from institutions involved in rehabilitation across sectors in the delivery system was a common organizational strategy across the municipal projects. Besides the obvious advantage of cross-disciplinary assessment of cases, the formal mixed steering committees functioned as ‘door openers’ in regard to the various levels and agents of rehabilitation activities. For instance, leaders both in hospitals and community departments who were on steering committees would introduce rehabilitation projects at department meetings to facilitate cooperation by their staff. Another example was noted by participants who explained that representatives from different disciplines could more easily establish collaboration with other departments if they knew people in those departments from their own profession.

Another important dynamic was the power relationship between hospitals and community-based services. RMs suggested that the hospital staff had low expectations of community-based services. In one of the projects, a local project leader conducted a few pilot interviews with hospital staff who distinctly expressed mistrust in community-based services being able to fulfill the task of providing rehabilitation. Therapists in the hospital did not expect that health care staff in the community could deliver an equally good intervention despite their shared professional background. These problems were in part related to the delivery context and the values and beliefs that go with them--the care cultures operating in municipalities; e.g., hospital services are to cure disease, and communities’ efforts are to support functioning in everyday life.

Despite relationships, steering committees, and attempts to promote collaboration across professions and delivery systems involved in rehabilitation, communication for coordination purposes seemed to be an ongoing challenge in projects. In four of the municipalities, RMs reported that they used the International Classification system of Functioning (ICF) to providing a platform for mutual ‘language’ and shared understanding. One municipality in particular invested extra effort in disseminating knowledge to health care staff involved at all levels of rehabilitation by providing a scenario of continuing education seminars as part of their project.

### Insufficient pathways and collaborative keys to rehabilitation

As alluded to above, the data brought attention to collaboration and insufficient pathways as prime factors for coordination of rehabilitation services as well as for making the course of rehabilitation coherent. Moreover, rehabilitation managers from multiple disciplinary backgrounds emphasized communication including referral pathways of patients from one delivery system to another as barriers for establishing rehabilitation while they pointed to collaboration and visibility strategies to overcome the problems encountered.

### Barriers for community-based cancer rehabilitation

Inclusion of cancer-survivors to community-based rehabilitation was affected by the way in which referrals from hospital and community-based services were organized. In Denmark patients who transfer from hospital to community-based services are supposed to be assessed for rehabilitation needs at the hospital. The patient’s oncologist can refer the patient to the community-based services, but referral to those services is not consistently provided by all hospitals and not for all cancer diagnosis.

All of the RMs reported that despite intensive efforts to include cancer patients in need of rehabilitation, referrals were rarely received. The RMs therefore engaged in comprehensive and time-consuming activity to inform cancer survivors about rehabilitation options. Furthermore, the RMs reported that the majority of clients enrolled in the community-based rehabilitation programs were dominated by resourceful cancer survivors (often women with breast cancer). Cancer survivors who were either socially deprived (e.g., low income, unemployed, on pension), males, or people with a non-Danish ethnic background were hardly represented among the client group. These inclusion problems brought RMs’ attention to two central issues: referral procedures and equality of access for all cancer survivors. RMs therefore developed strategies and recommendations to overcome these issues.

### Strategies for equal inclusion

One of the primary strategies that RMs devised to increase inclusion in community-based rehabilitation was identification of cancer survivors while they were still in hospital. RMs recommended an initial contact at the time of diagnosis with an in-person meeting between a community-based RM and the cancer patient. RMs also suggested the alternative approach of initiating contact during early stages of treatment. The purpose of this type of intervention is to reach the patient after the shock of diagnosis but while increased attention to life at home has happened. Furthermore the RMs proposed local visibility and easy access to community-based services. For example one of the projects had a rehabilitation bus that was parked in different public places of the municipality to make the rehabilitation and survivor service well known among community citizens. In addition, RMs experiences from the projects revealed that socially deprived citizens could more easily access services when these were located in the vicinity of public transportation.

As another strategy, one project developed successful collaboration with General Practice (GP) of the area through close collaboration with a member of the local GP network. This led to a raise in referrals from GPs’ to the community-based cancer rehabilitation project. Finally to bridge the gap and transition from hospital to community-based rehabilitation, the RMs proposed to base coordination on clear collaboration agreements between hospital and community-based services. Such experimentation with and refinement of novel approaches seemed to improve collaboration among rehabilitation providers and increase referral of cancer survivors.

Once cancer survivors were included to rehabilitation the challenge was to coordinate the array of services needed for the individual as well as creating optimal coherence in the course of rehabilitation. Also in this endeavor collaboration was identified to be the key. Moreover, the RMs all found that realistic rehabilitation plans was depending on close collaboration with the individual cancer survivor. To enhance coherence in the cancer survivors rehabilitation process the RMs experienced that a minimum of three rehabilitation in-person meetings was required. One should be held as an initiating meeting based on motivational principles, to support the cancer survivor’s personal resources and for empowerment purposes. A second in-person meeting was recommended by the RMs to evaluate and if necessary adjust initial goals for the rehabilitation process. Finally the third in-person meeting should be to provide a future oriented evaluation.

### Advantages of team-based rehabilitation

In addition to the challenges of identifying and including cancer survivors in the community-based rehabilitation programs the municipality projects had to develop ways to organize their services. In particular, team-based organization was identified to be of advantage both for collaborative purposes as well as for the coordination of and coherence in the cancer survivors’ rehabilitation course.

### Two ways of organizing rehabilitation-management

**Figure 1:**
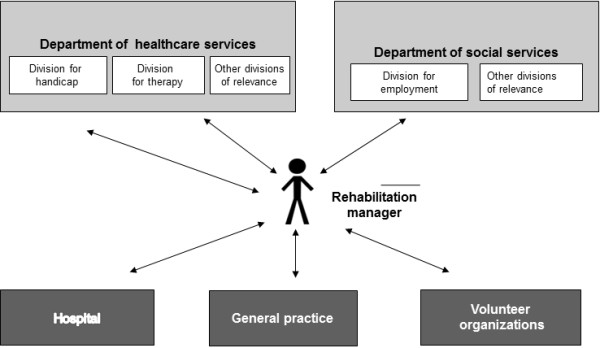
Non-team/single profession approach.

**Figure 2:**
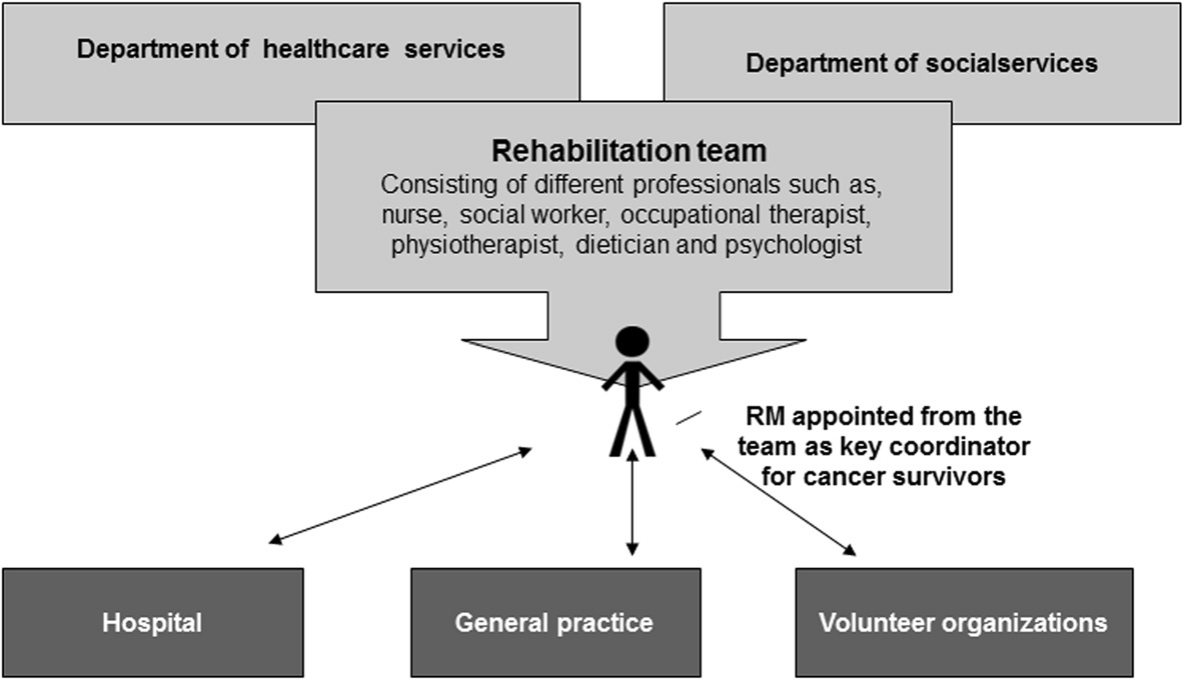
Team based/Inter-professional approach.

Different ways of organizing the case management were represented in the 15 municipalities, and these turned out to have very different implications for the rehabilitation programs over time. Twelve municipalities based the coordination of rehabilitation on non-team approach (see Figure 1). A non-team approach was either a single RM, or on 2 or more RMs with the same professional background, (identified hereafter as a ‘single-profession approach’), employed in the community either in the department of social services (two places) or in the department of healthcare services (ten places). Three municipalities used a team-based (inter-professional) approach to rehabilitation coordination (see Figure 2). Both models of rehabilitation coordination appointed a personal RM (key coordinator) to enhance coherence of the rehabilitation process for the individual cancer survivor. The personal RM could be anyone among the rehabilitation team and would often be allocated according to the main problems of the cancer survivor.

Based on focus group discussion the RMs pointed out we found that coordination based on a single RM required that the RM had extensive contacts to all rehabilitation service providers. The findings showed that whether the RM was situated within the social or the healthcare service department of the municipality had significantly different implications for care. Influenced in part by issues of culture and context as presented above, the immediate and ‘natural’ collaborators consisted of different professionals and the overall scope of rehabilitation varied accordingly, depending on the department. For example, when rehabilitation was provided from the social service department, rehabilitation focused on cancer survivors’ return to work. In that setting, staff primarily included social workers and legal advisors. Whereas when rehabilitation was provided from the healthcare department, the focus was primarily on recovery for disease and management of everyday life in a broader sense--including self-care at home, family relations and social network. The staff in this department had a background in healthcare disciplines such as nurses, psychologists, physiotherapists, occupational therapists, and dieticians.

Our study participants shared that a single-profession approach to rehabilitation management required the individual RM to ascertain significant in-depth knowledge of a wide variety of other fields. Moreover, to coordinate and guide the cancer survivor to appropriate services it was necessary to have knowledge of, for example, both social welfare regulations as well as disease specific knowledge. RMs suggested that these were significant obstacles to better cancer rehabilitation care.

As an example from the focus group reflections on organization of rehabilitation management, one of the RMs said;

”Although it is not possible to work team-based at the present time in our Municipality, we are working on establishing a modified team with representatives from relevant services such as the job center, general practice and the hospitals. Maybe it does not have to be a permanent team, with representatives from all areas at each meeting, but it should be possible to gather part of the team for specific problem matters. It should, however, always be the same person from each service area so it is clear who should be contacted for the actual problem and so the person has experience with the work procedures in the team and the community rehabilitation program (Belinda).

In response to Belinda – Peter from another Municipality contributed:

In Sellow Municipality we have success with such an adhoc model. But all team-members are present at all meetings.

Through the analysis of the data from the different projects and the focus group discussions we identified the team-based model to hold several advantages. This model seemed to provide optimal conditions for developing coordination of services delivered in concert by multiple providers within the community. First, the RMs involved in teams reported that there was a high degree of knowledge exchange among team professionals. This exchange gave all team members in-depth knowledge of the others’ professional competencies. That in turn made it easier for the individual RM to refer the cancer survivor to other interventions by other team members. Second, the team-based model was identified by the RMs to promote competence merging, in so far that team members could cover cases for each other (within what was professionally acceptable). For example, RM, Margaret from one of the larger Municipalities shared,

The team-based approach at our place has led to more fluent boundaries among the team. So for instance the social worker sometimes covers for our psychologist in providing supportive conversations and then if the problem is more persisting she refers to the team psychologist in case needed.

Third, the team-based model supported an interdisciplinary approach to rehabilitation, increasing a mutual understanding among the team professions that in turn also seemed to support integration of services to the clients. Finally, the team-based model was found to be less vulnerable to issues such as staff shortages caused by, for example, sick leave or on other occasions.

## Discussion

This study focused on community-based rehabilitation for cancer survivors in the Danish context, and it revealed significant health service issues related to the coordination and coherence of care. Coordination and coherence are concerns in a horizontal structure, such as among the services offered within an on-the-ground community care system. They also are concerns in the vertical articulation of systems, such as between a hospital system of services and on-the-ground community services. An array of professional services, both across horizontal and vertical structures, was involved in a community-based cancer rehabilitation project in Denmark. Rehabilitation managers participating in this study indicated that the encounters of various health-care cultures--both among different professions and across different delivery systems, hospital and community-based--was a key issue for the delivery of rehabilitation services. Furthermore, the RMs noted that for various reasons, many cancer survivors were not receiving needed services. In particular they identified unequal inclusion condition for cancer survivors with regard to gender, ethnicity, and socio-demographic background. Similar challenges such as organization of rehabilitation and equal inclusion are reported from research about community cancer rehabilitation around the world and highlight the need for organizational as well as procedural strategies to solve these problems [18,19].

In response to the issue of inclusion, RMs proposed that cancer survivors should be initially contacted by community-based rehabilitation as early as the time of diagnosis. This suggestion may not be the most appropriate for cancer-survivors who at the time of diagnosis may be in a state of shock and not oriented towards the future of returning to life in the community. Rather, the suggestion may be seen as an indication of the lack of a well-developed referral system, securing equal opportunities for rehabilitation for all cancer survivors identified to have rehabilitation needs. The results also suggest that barriers for developing coordination and coherence were in part due to the lack of prior cooperation across groups as well as insufficient knowledge among professions of discipline related competencies.

The identified cultural barriers within systems and among rehabilitation professionals draw attention to underlying factors which may be pertinent to secure the overall quality of coordination and coherence in rehabilitation. From an organizational perspective, the development of increased dialogue between providers can make cultural barriers visible and raise awareness of existing and tacit knowledge supporting possibilities for change. By extension, if we relate the cultural barriers to Leavitt’s system oriented model [20], our findings suggest that culture is shaped by, and dependent on, several variables, including existing technology, organizational structure, and the people involved. Collaboration, and therefore coordination and coherence, can be influenced by the particular combination of organization and types of professions involved. Data from our municipalities and RMs suggest that the particular mix in each place had an effect on how cultures were formed, how they worked, and how rehabilitation was provided.

Based on the results, it appears necessary that rehabilitation staff within and across providers share a mutual language and understanding. The ICF may be a potential means for such purposes and for bridging cultural differences. Generally the ICF has received much attention in Scandinavia as a common conceptual classification system useful for rehabilitation and interdisciplinary collaboration [16,21-23]. However, drawbacks of the ICF have been highlighted, including unqualified differentiation of main terms such as activity and participation [23,24]. The RMs in this study found the ICF useful, and we suggest that further studies of the potentials and consequences of ICF use in community-based cancer rehabilitation are needed.

The team-based model for rehabilitation case-management offered several advantages for coordination and coherence. This solution may not be an option for all municipalities, however, because of population, geography, and available RMs. Small communities may have to develop other approaches, such as team-based services that are not diagnosis specific. The results also showed that rehabilitation managers can come from a variety of professional backgrounds and should not be limited to a single profession. Required general competencies for community-based RMs included certain cancer specific knowledge integrated with a focus supporting cancer survivors in returning to contexts of everyday living.

The complexity of organizations within health care systems, including hospitals and local provider groups, are often so complex that no single analytical tool can capture it adequately. Furthermore, organizations of health care services are pluralistic in form and in constant evolution. Because organizations are continuously subject to political shifts and other changes specific to time and place, approaches to community-based cancer rehabilitation must be based in principles that provide flexibility. In that light, the present study does not intend to suggest ‘final’ solutions to cancer rehabilitation but rather to reveal possibilities that can guide cancer care providers and organizations.

## Conclusion

The findings of this study extend existing knowledge by using the experiences of various professionals involved in establishing community-based cancer rehabilitation. Coordination and coherence in community-based rehabilitation relies on increased collaboration facilitated by informal relationships and formal networks across disciplines and delivery systems. Communication and common understanding supported by use of shared frameworks within and across systems can enhance rehabilitation services. While there is much yet to be learned about the relatively new move to community-based cancer rehabilitation, the experience from Danish municipalities points to key strategies that may improve coordination and coherence of care in other contexts.

## Abbreviations

RM: Rehabilitation Manager; GP: General practice; ICF: International classification system of functioning.

## Competing interests

Both authors declare no conflict of interest and the authors alone are responsible for the content of the paper.

## Authors’ contributions

The study was led and conducted by the first author, KlC. KlC performed the main analysis, which was further developed and critically revised in close cooperation with the second author, MC. Both authors approved the final manuscript.

## Authors’ information

MPC, Ph.D. is Professor and Chair, Department of Health Care Sciences, Eugene Applebaum College of Pharmacy and Health Sciences, Wayne State University, USA. KlC, Ph.D. is Associate professor and Head of the Research initiative of Activity Studies and Occupational Therapy at the Research unit Health, Man and Society, Institute of Public Health, University of Southern Denmark, DK.

## Declarations

This manuscript has not been submitted elsewhere and the authors declare no conflicts of interest with respect to the authorship and/or publication of this article.

## Pre-publication history

The pre-publication history for this paper can be accessed here:

http://www.biomedcentral.com/1472-6963/13/339/prepub
